# Alkaline shock protein 23 (Asp23)‐controlled cell wall imbalance promotes membrane vesicle biogenesis in *Staphylococcus aureus*

**DOI:** 10.1002/jev2.12501

**Published:** 2024-08-28

**Authors:** Jia Li, Keting Zhu, Chao Li, Wei Huang, Xing Tian, He Yan, Yan Zhao, Jing Zhou, Xindi Gao, Xiancai Rao, Gang Li, Renjie Zhou, Ming Li

**Affiliations:** ^1^ Department of Emergency Medicine the Second Affiliated Hospital of Army Medical University Chongqing China; ^2^ Department of Microbiology, College of Basic Medical Sciences, Key Laboratory of Microbial Engineering Under the Educational Committee in Chongqing Army Medical University Chongqing China

**Keywords:** Asp23, membrane vesicles, proteomic analysis, SigB, *Staphylococcus aureus*

## Abstract

Membrane vesicles (MVs) are produced by species across all domains of life and have diverse physiological functions as well as promising applications. While the mechanisms for vesiculation in Gram‐negative bacteria are well‐established, the genetic determinants and regulatory factors responsible for MV biogenesis in Gram‐positive bacteria remain largely unknown. Here, we demonstrate that a Q225P substitution in the alternative sigma factor B (SigB) triggers MV production in *Staphylococcus aureus* strain Newman by hindering the specific binding of SigB to the *asp23* promoter, thereby repressing expression of alkaline shock protein 23 (Asp23). Isogenic deletion of *asp23* also promotes MV formation in Newman, confirming the critical roles played by *sigB* and *asp23* in modulating *S. aureus* vesiculation. While bacterial growth and cytoplasmic membrane fluidity are not impaired, mutation of *asp23* weakens the cell wall and enhances autolysis, consistent with decreased expression of alpha‐type *psm* and *lrgAB* that modulate murein hydrolase activity. TEM and proteomic analysis show that Newman and *asp23* deletion mutant generate MVs with nearly identical morphology and composition, but virulence‐associated factors are significantly enriched in MVs from the *asp23* mutant. Overall, this study reveals novel genetic determinants underlying *S. aureus* vesiculation and advances the understanding of the physiology of MV biogenesis in *S. aureus*.

## 1 INTRODUCTION

Extracellular vesicles, also known as membrane vesicles (MVs), are nano‐scale, spherical, bilayered structures produced by species across all three domains of life (prokaryotes, eukaryotes and archaea) (Deatherage & Cookson, 2012). Similar in structure to live bacteria, MVs carry a diverse payload of proteins, nucleic acids and lipids that can function in bacterial communication, nutrient acquisition, DNA transfer, biofilm formation, antibiotic resistance, immune modulation and pathogenesis (Toyofuku et al., 2023). In addition to these multifaceted physiological roles, MVs are now attracting attention as potential infectious biomarkers, bacterial vaccines, cancer immunotherapy agents and drug delivery platforms (Gan et al., 2023).

Vesiculation is a complex process, and two primary mechanisms have been identified in Gram‐negative bacteria to date (Toyofuku et al., 2023). One involves the blebbing of membrane materials from living cells, resulting from cell envelope disturbances such as imbalanced peptidoglycan biosynthesis, the accumulation of misfolded proteins in the periplasmic space, or the intercalation of hydrophobic molecules into the outer membrane, yielding ‘classic’ MVs (B‐type MVs). The second route is the result of explosive cell lysis triggered by genotoxic stress that activates the expression of prophage‐derived endolysins, causing degradation of the peptidoglycan layer and cell lysis. Self‐assembly of the shattered membrane components subsequently generates ‘explosive’ MVs (E‐type MVs).

In contrast to Gram‐negative bacteria, Gram‐positive bacteria were long thought to be incapable of producing MVs because of their thick cell walls. Evidence for MVs secreted by Gram‐positive bacteria was first reported in 1990 (Dorward & Garon, 1990), but direct demonstration of vesicle formation via transmission electron microscopy (TEM) and proteomics was not reported until 2009 (Lee et al., 2009). In analogy to explosive cell lysis in Gram‐negative bacteria, Gram‐positive bacteria can also protrude their cytoplasmic membrane through holes in the peptidoglycan layer that prophage‐encoded endolysins have generated. The externalized membranes are then released as explosive cytoplasmic membrane vesicles (ECMVs) (Toyofuku et al., 2019). However, unlike explosive cell lysis in Gram‐negative bacteria, Gram‐positive cells are not completely lysed during ECMV biogenesis but instead form ghost cells. This process, subsequently named ‘bubbling cell death’, has been documented in several Gram‐positive species, including *Bacillus subtilis* (Toyofuku et al., 2017), *Mycobacterium tuberculosis* (Lee et al., 2015), *Listeria monocytogenes* (Karthikeyan et al., 2019), *Streptococcus suis* (Haas & Grenier, 2015) and group A *Streptococcus* (Resch et al., 2016). Nonetheless, understanding of the biogenesis and genetic regulation of MVs in Gram‐positive bacteria is still in its infancy and lags behind that of MVs in Gram‐negative bacteria.

*Staphylococcus aureus*, a source of dangerous infections throughout the world, has been widely used as a model organism to study the biogenesis of MVs in Gram‐positive bacteria. In addition to the bubbling cell death mechanism described above, vesiculation in *S. aureus* is also proposed to occur through a blebbing mechanism in which disruption of the cytoplasmic membrane by amphipathic phenol‐soluble modulins (PSMs) is followed by secretion of MVs through the cell wall after it has been weakened by peptidoglycan‐lytic autolysins (Wang et al., 2018). In support of this model, deletion of the major autolysin‐coding genes *sle1* and *atl* reduce the release of *S. aureus* MVs, consistent with their roles in peptidoglycan degradation (Wang et al., 2018). Recently, the delta‐hemolysin Hld was demonstrated to modulate vesiculogenesis and influence the properties of *S. aureus* MVs (Chen et al., 2023; Wang et al., 2023). However, the genetic determinants and underlying mechanisms that control MV biogenesis and cell wall transit in *S. aureus* remain largely unknown.

Previously, we identified a point mutation (Q225P) in the alternative sigma factor B (SigB) that triggers MV production in *S. aureus* strain Newman, suggesting that SigB has an important regulatory role in *S. aureus* MV biogenesis (Qiao et al., 2022). In this study, we further investigated the underlying cues responsible for the formation of MVs controlled by SigB. We discovered that the Q225P substitution hinders the binding of SigB to the *asp23* promoter, resulting in a significant reduction in the expression of alkaline shock protein 23 (Asp23). Deletion or down‐regulation of *asp23* results in a thinner cell wall and enhances cell autolysis, ultimately causing hypervesiculation in *S. aureus*. Overall, this study unravels a novel pathway for the biogenesis of *S. aureus* MVs and improves our understanding of the physiology of MV release in *S. aureus*.

## 2 MATERIAL AND METHODS

### 2.1 Bacterial strains and culture conditions

Bacterial strains and plasmids used in this study are listed in Table S1 in the Supplemental material. The reference *S. aureus* strain RN4220 was a gift from Prof. Baolin Sun (University of Science and Technology of China, China), and the Newman strain was kindly provided by Prof. Lu Yu (Jilin University, China). Unless other specified, *S. aureus* strains were cultivated in brain heart infusion (BHI) medium (Oxoid, UK) at 37°C with shaking (200 rpm) or grown on BHI agar. The *Escherichia coli* strain Trans1‐T1 (Transgen, China) was cultured in Luria–Bertani (LB) medium (Oxoid, UK) at 37°C with shaking or grown on LB agar. For maintenance of plasmids pBT2, pLI50, pXR and derivatives (Table S1), cultures were supplemented with 100 μg/mL of ampicillin for *E. coli*, and 10 μg/mL of chloramphenicol for *S. aureus*.

### 2.2 Construction of gene deletion mutant and complementary strain in *S. aureus*

Gene allelic deletion and complementation were performed in *S. aureus* as previously described with minor modifications (Rao et al., 2022). To knock out *asp23*, the left flanking region (Up‐*asp23*) and right flanking region (Down‐*asp23*) adjacent to *asp23* were amplified from genomic DNA (gDNA) using the primer pairs listed in Table S2 in the Supplemental material. A fusion fragment was generated by overlapping PCR using the Up‐*asp23* and Down‐*asp23* fragments as templates, then ligated into the temperature‐sensitive shuttle vector pBT2 (previously digested with *Xba* I/*Eco*R I) using Gibson assembly master mix (NEB, USA). The resulting plasmid was designated as pBT2∆*asp23*. This plasmid was transformed into *S. aureus* strain RN4220 for modification and subsequently electroporated into strain Newman (designated NM in this study), and into strain NM containing the Q225P mutation in SigB (designated NMQ). *S. aureus* cells were cultivated at 42°C for the integration of plasmid pBT2∆*asp23* into the bacterial genome via single cross‐over events, followed by plasmid excision via double cross‐over events at 25°C growth. The double cross‐over mutants that are sensitive to chloramphenicol were further confirmed by PCR and sequencing for the seamless deletion of *asp23*.

For genetic complementation, a fragment containing the promoter region and coding sequence of *asp23* was amplified from strain NM using the appropriate primers (Table S2) and then cloned into shuttle plasmid pLI50 using Gibson assembly master mix (NEB, USA) to generate pLI*asp23*. Finally, pLI*asp23* was introduced into the mutant strain NM∆*asp23* and NMQ, generating the complementary strains NM∆*asp23*/*asp23* and NMQ/*asp23*, respectively. The empty vector pLI50 served as a negative control. For overexpression of alpha‐type PSMs and LrgAB in the *asp23* deletion mutant, the genetic operon alpha‐type *psm* (comprising *psmα1*, *psmα2*, *psmα3* and *psmα4*) and the dicistronic operon *lrgAB* (containing *lrgA* and *lrgB*) was fused to a xylose‐inducible promoter using the expression vector pXR, resulting in pXR*psmα* and pXR*lrgAB*, respectively. Overexpression of *psmα* and *lrgAB* was induced by the addition of xylose to a final concentration of 0.5% (wt/vol) during bacterial growth.

### 2.3 Growth measurement

*S. aureus* strains of interest were grown overnight in BHI with shaking at 37°C and diluted 1:1000 into fresh BHI medium. Aliquots of 200 μL were inoculated into a 96‐well flat‐bottomed plate (Corning, USA) with three replicate wells for each strain and cultivated at 37°C for 24 h. Optical density at 600 nm was measured every hour after inoculation and plotted versus culture time to generate growth curves.

### 2.4 Preparation and quantification of *S. aureus* MVs

MVs were recovered from *S. aureus* as previously described (Yuan et al., 2018). Briefly, overnight cultures of *S. aureus* strains of interest were inoculated into 1 L of BHI broth (1:100) and incubated for 16 h at 37°C with shaking at 200 rpm. Culture supernatants were collected by centrifugation at 10,000 × *g* at 4°C for 30 min to sediment cells, and then filtered through 0.22 μm Millex syringe filters (Beyotime, China) to eliminate any remaining cell debris. The filtered supernatants were subjected to centrifugation at 200,000 × *g* at 4°C for 3 h, and the MV pellets were washed twice with phosphate‐buffered saline (PBS, pH 7.2). To remove membrane fragments and protein aggregates, the crude MVs were resuspended in 4.4 mL of 50% Optiprep buffer (STEMCELL, Canada), and transferred to an ultracentrifuge tube. Carefully, 4 mL of 40% Optiprep gradient buffer and 1.6 mL of 10% Optiprep gradient buffer were sequentially added to form distinct upper layers. The tube was centrifuged horizontally at 200,000 × *g* at 4°C for 3 h, after which the MVs were collected at the interface between the 40% and 10% layers. The MVs were dissolved in PBS and stored at −80°C.

For quantitation, MV proteins were separated by 12% SDS‐PAGE, stained with Coomassie Brilliant Blue R‐250 (Thermo Scientific, USA) and photographed. MV protein concentration was determined using an enhanced BCA Protein Assay Kit, following the recommended procedures (Beyotime, China). Lipid content was quantified using the membrane‐specific fluorescent lipophilic dye FM4‐64, according to the suggested protocol (Invitrogen, USA). The MV particle number was determined using nanoparticle tracking analysis.

### 2.5 Scanning electron microscopy (SEM) and transmission electron microscopy (TEM)

Morphology and structure of *S. aureus* cells were observed using SEM and TEM. For SEM observation, stationary‐phase (16 h) cultures of *S. aureus* were harvested by centrifugation at 10,000 × *g* for 5 min at 4°C, then rinsed three times with PBS. The bacterial pellets were first fixed with 2.5% glutaraldehyde for 6 h, dehydrated using a gradient of ethanol concentrations (30%, 50%, 70%, 90% and 100%) for 10 min each, then resuspended in isoamyl acetate to replace the ethanol. Finally, 10 μL of the bacterial suspension was dropped onto a silicon wafer, volatilized and dried at 37°C. Samples were sprayed with gold and observed by SEM (FEI NanoPorts, Japan).

For TEM analysis, cryosections of fixed stationary‐phase (16 h) *S. aureus* cells were stained and stabilized with 2% methylcellulose (25 cps) containing 0.3% uranyl acetate, and then observed by TEM (JEM‐1400FLASH, Japan). To observe MV particles, purified MVs were placed on 230‐mesh formvar/carbon‐coated copper grids (Zhong Jing Ke Ji Tech, China) and negatively stained with 2% (m/v) uranyl acetate for 15 s. Electron micrographs were recorded using a JEM1011 microscope (JEOL, Japan) at an acceleration voltage of 100 KV. Morphometric evaluation of cell wall thickness was performed as previously described (Cui et al., 2000). The thicknesses of the cell wall of 30 cells of each strain were measured at 10 different points with nearly equatorially cut surfaces.

### 2.6 SDS‐PAGE and Western blot analysis

Protein samples were dissolved in 5×SDS‐PAGE buffer and denatured at 100°C for 10 min. After centrifugation, aliquots of supernatant were separated using 12% SDS‐PAGE and imaged after staining with Coomassie Brilliant Blue R‐250 (Thermo Scientific, USA). For Western blotting, proteins were transferred from the acrylamide gels to PVDF membranes (Beyotime, China). Membranes were blocked using 5% (m/v) skim milk in high‐salt Tris‐buffered saline (HS‐TBS, 20 mM Tris, 500 mM NaCl, pH 7.5) for 1 h at room temperature. Membranes were then incubated overnight at 4°C in a 1:10,000 dilution of rabbit anti‐Asp23 antibody (Sangon Biotech, China). After washing five times with PBS‐T (PBS containing 0.05% Tween‐20), membranes were treated with 1:10,000 goat anti‐rabbit IgG‐horseradish peroxidase conjugate (Solarbio, China) for 1 h at 37°C. Protein bands were visualized using Pierce ECL Western Blotting Substrate (Thermo Scientific, USA) and photographed using a ChemiDoc XRS Imaging System (Bio‐Rad, USA).

### 2.7 Electrophoretic mobility shift assay (EMSA)

EMSA assays were performed as previously described (Zheng et al., 2022). Briefly, a biotin‐labelled DNA probe containing the *asp23* promoter region was amplified by PCR from NM gDNA using a 5′‐biotin‐labelled primer (Table S2), and then incubated with purified recombinant His‐tagged SigB and SigB(Q225P) proteins in EMSA/Gel‐Shift binding buffer (Beyotime, China), following the manufacturer's instructions. After incubation at 25°C for 20 min, the mixture was separated in a 6% native polyacrylamide gel at 100 V and then transferred to a nylon membrane in 0.5 × trisborate‐EDTA (TBE) buffer at 380 mA for 30 min. The biotin‐labelled DNA fragments were cross‐linked using a UV cross‐linker (SCIENTZ, China) at 120 mJ/cm^2^ for 60 s, and detected using a chemiluminescent nucleic acid detection module kit (Thermo Scientific, USA) following the recommended protocol. Bands were imaged using a Fusion Pulse imaging system (VILBER, France). Unlabelled probes were added in 200‐fold excess as specific competitors, and the biotin‐labelled probes containing the mutated SigB binding site or the *saeR* promoter region served as negative controls. To quantitatively compare the binding capacity between SigB and SigB(Q225P), the intensity of the unbound probe was quantified using ImageJ software (ImageJ software, USA), and the intensity of the free probe in the absence of protein was set to the amount of total probe. Then, the fraction of bound probe was calculated as ([total probe—unbound probe]/total probe).

### 2.8 Construction of the *asp23* promoter reporter strain

The promoter region of *asp23* was amplified by PCR using gDNA from the strain NM and the appropriate primers (Table S2). The product was ligated into vector pGFP (pre‐digested with *Eco*R I and *Bam*H I), yielding pGFP*asp23*, in which the expression of the green fluorescent protein is under the control of the *asp23* promoter. The recombinant plasmid was transformed into the NM and NMQ strains, generating the reporter strains NM/pGFP*asp23* and NMQ/pGFP*asp23*, respectively. The activity of the *asp23* promoter was detected by measuring fluorescence intensity, and values were normalized using the OD600 values of the corresponding bacterial cultures.

### 2.9 Triton X‐100‐induced autolysis assay

Autolysis assays were performed as previously described with minor modifications (Shu et al., 2023). Bacterial cultures were incubated overnight, then used to inoculate (1:100) fresh BHI and cultured at 37°C with shaking for 16 h. Cells were collected by centrifugation at 5000 × *g* for 3 min and washed twice with 50 mM Tris‐HCl buffer (pH 7.5). The pellets were resuspended in the same buffer containing 0.05% (vol/vol) Triton X‐100 to an OD600 of 0.8, and incubated at 37°C with shaking at 200 rpm. Lysis was determined by monitoring the progressive decrease in OD600 values at 30 min intervals using a microplate reader (Bio‐Tek, China).

### 2.10 Cell membrane integrity and fluidity analysis

Cell membrane integrity was assessed using the fluorescent dye propidium iodide (PI), which can only penetrate bacteria with damaged cell envelopes. Overnight cultures of the strain NM and its derivatives were diluted 1:100 into fresh BHI and cultured at 37°C with shaking at 200 rpm. Cells were collected at 16 h by centrifugation at 5000 × *g* for 5 min, washed, and resuspended with sterile PBS. 10 μL PI solution (10 μg/mL) was added to the suspension, which was then incubated at 37°C for 30 min. Fluorescence was measured using the SmartSpecTM3000 spectrophotometer (Bio‐Rad, USA) with excitation and emission wavelengths of 520 and 627 nm, respectively. Bacterial suspensions pretreated with 1% Triton X‐100 for 1 h were used as a positive control.

Membrane fluidity was determined by the Laurdan fluorescence probe method (Weng et al., 2023). Briefly, overnight cultures were diluted 1:100 into fresh BHI and cultured for 16 h at 37°C with shaking at 200 rpm. Cells were collected by centrifugation at 5000 × *g* for 5 min, washed and resuspended in PBS. Laurdan solution was then added to a final concentration of 1 mmol/L, and the mixture was incubated at 37°C in the dark for 30 min. After centrifugation at 5000 × *g* for 5 min, fluorescence was measured using a SmartSpecTM3000 spectrophotometer (Bio‐Rad, USA) with excitation and emission wavelengths of 340 and 440/490 nm, respectively. Generalized polarization (GP) from emission spectra was calculated as previously described (Verstraeten et al., 2021).

### 2.11 Isolation and sequencing of total cellular RNA

Overnight cultures of the NM and mutant NM∆*asp23* strains were diluted 1:100 into fresh BHI and incubated at 37°C with shaking for 6 h. Log‐phase cultures were first lysed with lysostaphin (Sigma–Aldrich, USA), and total cellular RNA was isolated using a RNAprep Pure Cell/Bacteria Kit (Promega, USA) according to the manufacturer's protocol. After the assessment of RNA quality, library preparation and Illumina RNA sequencing were conducted by Shanghai Sangon Biotechnology (Shanghai, China). Differentially expressed genes (DEGs) were identified using the R package DESeq2 v1.12.4 (Love et al., 2014). Genes with fold change values ≥ 1.5 and with false discovery rate (FDR) *P*‐values < 0.05 were classified as differentially expressed. KEGG and COG enrichment analyses were performed using the R package clusterProfiler v3.0.5 with an adjusted *P*‐value < 0.05 (Wu et al., 2021).

### 2.12 RT‐qPCR analysis

Total RNA was isolated from stationary‐phase (16 h) cultures using the RNAprep Pure Cell/Bacteria Kit (Promega, USA) and reverse‐transcribed using a PrimeScript RT kit (TaKaRa, Japan), following the recommended methods. The cDNA was amplified using SYBR Ex Taq Master Mix (TaKaRa, Japan) and analyzed using a CFX96 Manager (Bio‐Rad, USA). Three biological replicates were performed for all RT‐qPCR assays. The primers used for quantification are listed in Table S2. Normalized gene expression levels were calculated using the 2^−ΔΔCT^ method, with *gyrA* expression as the endogenous control.

### 2.13 Proteomic analysis

Protein cargos carried by *S. aureus* strain NM (^NM^MVs) and mutant NM∆*asp23* (^∆^ *^asp23^* MVs) were analyzed using 4D‐label‐free liquid chromatography‐tandem mass spectrometry (LC‐MS/MS), performed by Jingjie PTM BioLab (Hangzhou, China). Briefly, ^NM^MV and ^∆^ *^asp23^* MV proteins (three biological replicates) were digested with trypsin, desalted on a Strata X SPE column, dissolved in 0.1% (vol/vol) formic acid and subjected to 4D‐label‐free LC‐MS/MS analysis. MS/MS data were processed using the Proteome Discoverer search engine (v.2.4). Tandem mass spectra were compared with *S. aureus* NM NCBI_AP009351.1_20230720_seqkit.fasta (2580 entries), concatenated with reverse decoy and contaminant databases. Trypsin (Full) was specified as the cleavage enzyme, allowing up to 2 missing cleavages. The minimum peptide length was 6, and the maximum number of modifications per peptide was 3. The mass error was 10 ppm for precursor ions and 0.02 Da for fragment ions. The FDR for protein, peptide and peptide spectrum matches was adjusted to < 1%.

The LC‐MS/MS data were normalized using the median centring method across total proteins to correct sample loading differences. All proteins were quantified with at least 2 unique peptides. The abundance of protein across all samples was centralized and transformed into their values of relative quantification in each sample. To adjust the systematic bias of the identified protein amount among different samples in the process of mass spectrometry detection, the relative quantitative value of protein was corrected by the median value. For differential expression analysis, the fold change was calculated by the ratio of the mean intensity for each protein in two sample groups, and the Student's *t*‐test was performed. The parameters for differential analysis were as follows: *P*‐value < 0.05, and the fold change > 1.5 or < 1/1.5 was regarded as differentially expressed proteins (DEPs).

The subcellular localization of identified proteins was predicted using the PSORTb, CELLO, AureoWiki and UniProt database. KEGG pathway annotation was performed using BLAST (blastp, evalue ≤ 1e‐4). For each sequence, annotation was based on the top‐scoring alignment. Fisher's exact test was used to analyze the significance of functional enrichment for DEPs. Functional terms with fold enrichments > 1.5 and *P*‐values < 0.05 were considered significant.

### 2.14 Statistical analysis

Unless specified otherwise, statistical analysis was performed using GraphPad Prism v8.0. Student's *t*‐test was used for comparison of two independent datasets. One‐way analysis of variance (ANOVA) or two‐way ANOVA was used to compare the means from multiple groups and assess the statistical significance. A *P*‐value less than 0.05 was considered significant.

### 2.15 Data availability

The raw RNA‐seq files have been deposited into the Gene Expression Omnibus (GEO) database under accession number GSE253193. All raw data of 4D label‐free LC‐MS/MS identification was deposited to the ProteomeXchange Consortium with the dataset identifier PXD048184.

## 3 RESULTS

### 3.1 The Q225P mutation in SigB triggers MV biogenesis by reducing *asp23* expression in *S. aureus*

**FIGURE 1:**
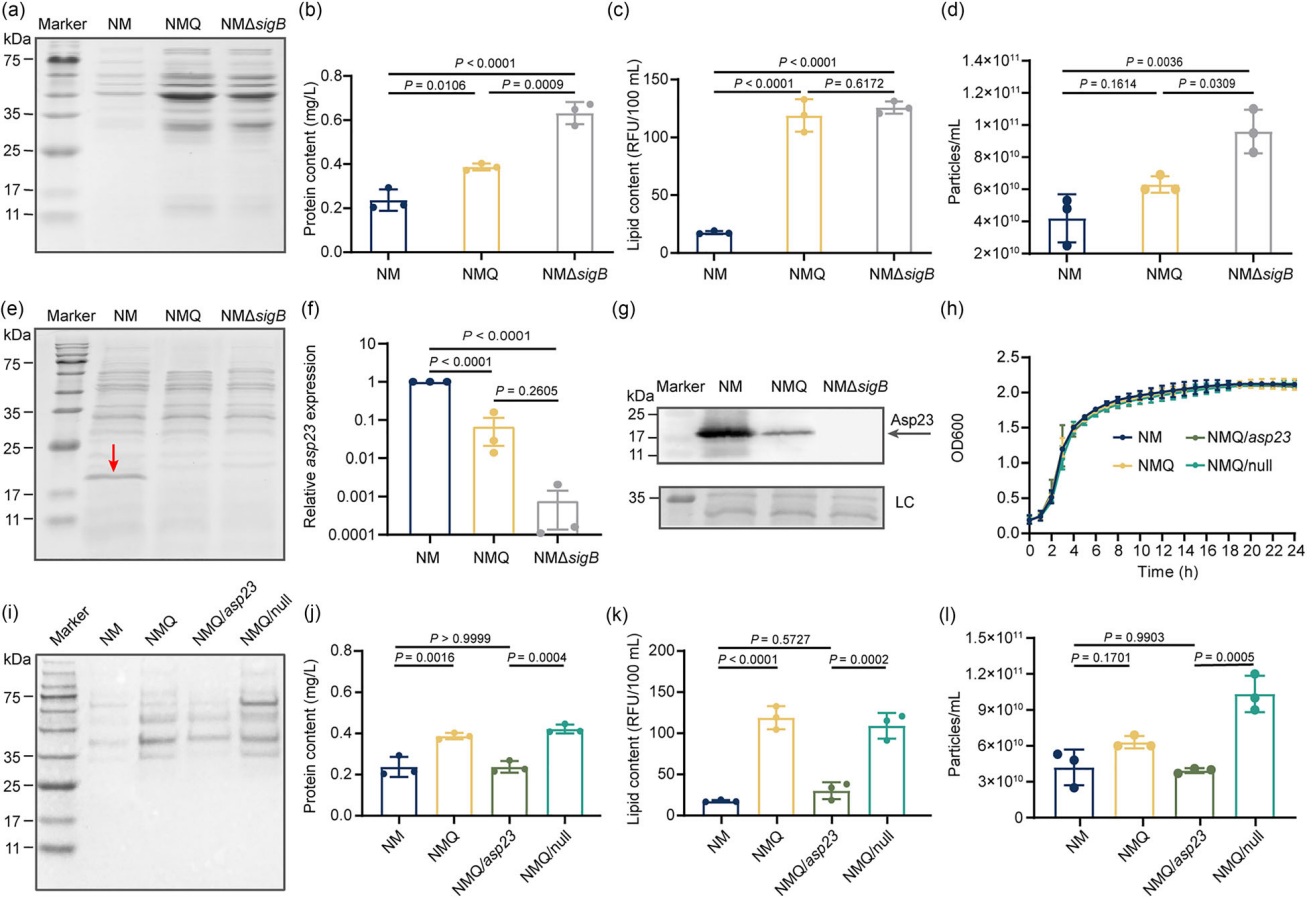
The Q225P mutation in SigB promotes MV production by repressing *asp23* expression. MVs derived from stationary‐phase (16 h) cultures of *S. aureus* strain NM, NMQ and NMΔ*SigB* were compared by (a) SDS‐PAGE analysis, and quantified for (b) protein content, for (c) lipid content, and for (d) MV particle numbers. Representative image for SDS‐PAGE is shown for three independent experiments. Data are expressed as mean ± standard deviation (SD) from three biological replicates and analyzed by one‐way ANOVA with Tukey's multiple comparisons test. (e) Whole‐cell protein extracts from *S. aureus* strain NM and derivatives were separated by 12% SDS‐PAGE. A band migrating between 17 and 25 kDa that is nearly absent in mutant NMQ and NMΔ*sigB* is indicated with a red arrow. Representative image for SDS‐PAGE is shown for three independent experiments. (f, g) Expression of *asp23* in NM, NMQ and NMΔ*SigB* was measured by (f) RT‐qPCR and (g) Western blot analysis. Data are expressed as mean ± SD of three biological replicates and analyzed by one‐way ANOVA with Tukey's multiple comparisons test. Representative gel for Western blot is shown from three independent experiments. LC: loading control. (h) Bacterial growth profiling. *S. aureus* strain NM and derivatives differing in *asp23* expression were cultivated in BHI broth in triplicate, and the optical density at 600 nm (OD600) for the cultures was monitored hourly for 24 h. (i–l) MVs recovered from stationary‐phase (16 h) cultures of NM, NMQ and complemented strains (NMQ/*asp23*, NM/null) were compared by (i) SDS‐PAGE analysis, and quantified for (j) protein content, for (k) lipid content, and for (l) MV particle numbers. Data are expressed as mean ± SD from three biological replicates and analyzed by one‐way ANOVA with Tukey's multiple comparisons test.

We reported previously that a Q225P substitution in SigB greatly stimulates MV production in *S. aureus* strain NM (Qiao et al., 2022). To confirm this phenotype, an isogenic deletion mutant of *sigB* was constructed in NM, namely NMΔ*sigB*. The MVs were then recovered from stationary‐phase (16 h) cultures using OptiPrep density gradient ultracentrifugation. SDS‐PAGE analysis revealed that both strain NMQ that harbours the Q225P mutation and strain NMΔ*sigB* produce obviously increased MVs than WT NM (Figure 1a). The enhanced yields of MVs were confirmed through the determination of MV protein and lipid content. While the WT strain released approximately 0.24 mg/L of MV‐derived proteins, NMQ and NMΔ*sigB* released 0.39 and 0.63 mg/L, respectively (Figure 1b). Measurement of the lipid content also revealed significantly elevated lipid production in both NMQ‐derived and NMΔ*sigB*‐derived MVs, in contrast to that of WT MVs (Figure 1c). In addition, direct quantitation of MV particle numbers by nanoparticle tracking analysis consistently demonstrated that increased MV particles were collected from both Q225P mutation and *sigB* deletion mutant, compared to WT (Figure 1d). These results unravel that SigB plays a critical role in modulating the biogenesis of MVs in *S. aureus*.

To investigate potential genetic factors involved in SigB‐regulated MV secretion, we initially extracted total cellular proteins from strain NM, NMQ and NMΔ*sigB*, respectively, and separated them using SDS‐PAGE. Similar patterns were observed in all three lanes, except that a band between 17 and 25 kDa was much less intense in NMQ and NMΔ*sigB* (Figure 1e). The corresponding band present in NM was then excised and analyzed by mass spectrometry. The protein was identified as alkaline shock protein 23 (Asp23) with a theoretical molecular size of 19.2 kDa. Further analysis using RT‐qPCR and Western blot confirmed that expression and production of Asp23 was significantly reduced in mutant NMQ, and almost undetectable in NMΔ*sigB* (Figure 1f,g).

To determine whether a decrease in Asp23 level is responsible for the increased production of MVs, the mutant NMQ was complemented *in trans* with a copy of *asp23* (the empty vector pLI50 was used as a control). The resultant strains were designated NMQ/*asp23* and NMQ/null, respectively. Growth kinetics in the complemented strains, NMQ, and WT were indistinguishable (Figure 1h). MV samples were then isolated, analyzed by SDS‐PAGE, and quantified for both protein and lipid contents, as well as MV particle numbers. The restoration of *asp23* expression greatly reduced MV production in NMQ/*asp23* (Figure 1i), yielding a protein level (0.24 ± 0.02 mg/L) comparable to that of the WT (0.24 ± 0.04 mg/L) (Figure 1j). In contrast, strain NMQ/null still exhibited enhanced MV production (Figure 1i), with protein levels (0.42 ± 0.02 mg/L) comparable to that of the mutant NMQ (0.39 ± 0.01 mg/L) (Figure 1j). Consistent with these results, analysis of both lipid content and MV particle numbers also demonstrated that complementation of *asp23* significantly decreased the release of MVs (Figure 1k,l), indicating that SigB(Q225P) promotes MV formation by reducing *asp23* expression.

### 3.2 The Q225P mutation impairs the binding of SigB to the *asp23* promoter

**FIGURE 2:**
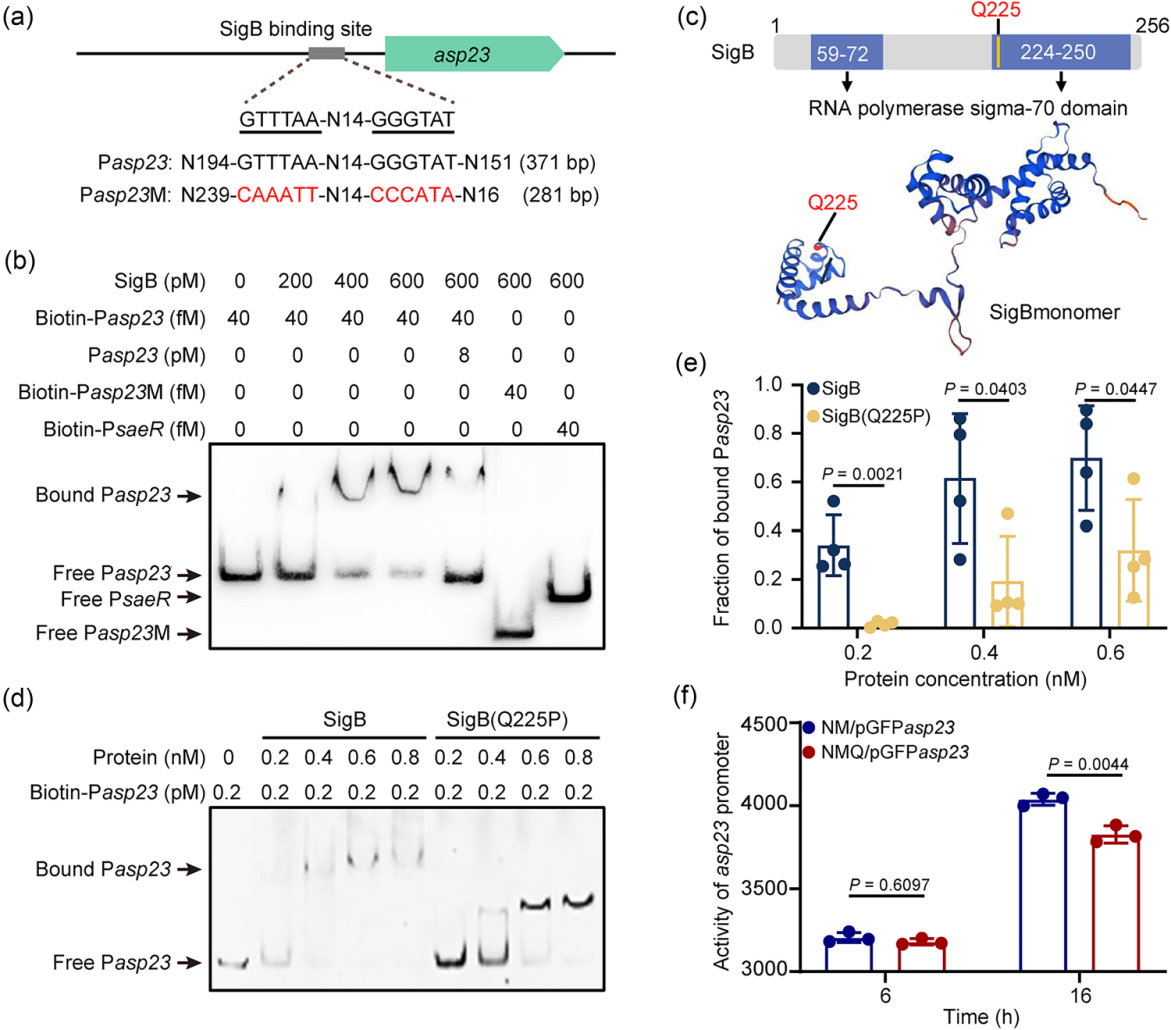
The Q225P mutation impairs binding of SigB to the *asp23* promoter. (a) The predicted SigB‐binding site in the *asp23* promoter. The probes used in the EMSA assay are indicated beneath with the mutated nucleotides highlighted in red. (b) EMSA analysis of specific binding of SigB to the *asp23* promoter. The biotin‐labelled probe (Biotin‐P*asp23*) was incubated with increasing concentrations of SigB (0 to 600 pM). A 200‐fold excess of unlabelled oligonucleotide (P*asp23*) was included as a competitor. A biotin‐labelled mutant probe (Biotin‐P*asp23*M) was used to assess binding specificity. Biotin‐labelled probes (Biotin‐P*saeR*) containing the *saeR* promoter region were used as a negative control. A representative gel from three independent experiments is shown. (c) Glutamine residue Gln225 of SigB is located within the predicted RNA polymerase sigma‐70 domain. (d) Comparing the binding capacity of protein SigB and SigB(Q225P) to probe Biotin‐P*asp23*. A representative gel from four independent experiments is shown. (e) The fraction of bound probe was calculated using the data in (d). The intensity of unbound probe was quantified using ImageJ, and the intensity of free probe in the absence of protein was set to the amount of total probe. The fraction of bound probe was calculated as ([total probe—unbound probe]/total probe). Data are expressed as mean ± SD from four biological replicates and analyzed by Student's *t*‐test. (f) Detection of *asp23* promoter activity. *S. aureus* strain NM and NMQ, both transformed with the GFP‐based reporter vector pGFP*asp23*, were cultivated in BHI. Fluorescence was measured for log‐phase (6 h) and stationary phase (16 h) cultures and normalized to bacterial growth (measured as OD600 values), respectively. Data are represented as mean ± SD from three biological replicates and analyzed by Student's *t*‐test.

The bacterial alternative sigma factor SigB regulates the stress response to changing conditions, and the expression of approximately 200 genes in *S. aureus*, can be directly and indirectly controlled by SigB (Jenul & Horswill, 2019). Previous studies have shown that *asp23* is regulated by SigB, and has been used as an indicator of SigB activity (Mitsuwan et al., 2019; Müller et al., 2014). In the promoter region of *asp23* in *S. aureus* NM, we identified a putative SigB‐binding site (5′‐GTTTAA‐N14‐GGGTAT‐3′) located 114 bp upstream of the start codon (Figure 2a). To examine the function of this region more closely, we generated and purified His‐tagged recombinant protein SigB and verified its binding to the putative binding site using an electrophoretic mobility shift assay (EMSA). The binding of SigB to the biotin‐labelled probes containing the predicted binding site affected migration in a dose‐dependent manner but was greatly diminished by the addition of a 200‐fold excess of unlabelled probes (Figure 2b). When the biotin‐labelled probe was mutated (5′‐CAAATT‐N14‐CCCATA‐3′) or substituted with a fragment encompassing the *saeR* promoter region (serving as a negative control), the binding and migration were completely abolished (Figure 2b), confirming the specificity for SigB that binds to the *asp23* promoter.

Given that the Q225P missense mutation occurs in the predicted RNA polymerase sigma‐70 domain in SigB (Figure 2c), we next determined whether the Q225P mutation affects the binding capability of SigB to the *asp23* promoter. EMSA analysis showed that the mutated protein SigB(Q225P) still retains the binding ability to the *asp23* promoter, but it is greatly impaired compared to that of the WT protein SigB (Figure 2d). Interestingly, the mutated protein SigB(Q225P) exhibited a different shift pattern with the faster migration and increased intensity of the SigB(Q225P)‐probe complex, as evidenced in the EMSA gel (Figure 2d). To quantitatively compare the binding capacity between SigB and SigB(Q225P), the intensity of the unbound probe (as indicated as a free probe in the EMSA gel) was first quantified, and the fraction of the bound probe was then calculated. The result revealed that the bound probes were significantly decreased for SigB(Q225P) in contrast to that of SigB (Figure 2e), demonstrating the impaired binding of SigB(Q225P) to the *asp23* promoter. Additionally, we constructed a GFP‐based reporter plasmid, in which the expression of GFP is directly controlled by the *asp23* promoter, and transformed them into the WT NM and mutant NMQ backgrounds. While no significant difference was detected between these strains in log‐phase (6 h) cultures, NMQ exhibited a significantly decreased *asp23* promoter activity compared to that of NM in stationary‐phase (16 h) cultures (Figure 2f). These findings indicate that the SigB(Q225P) reduces *asp23* expression by impairing the binding of SigB to the *asp23* promoter.

### 3.3 Isogenic deletion of *asp23* promotes MV formation in *S. aureus*

**FIGURE 3:**
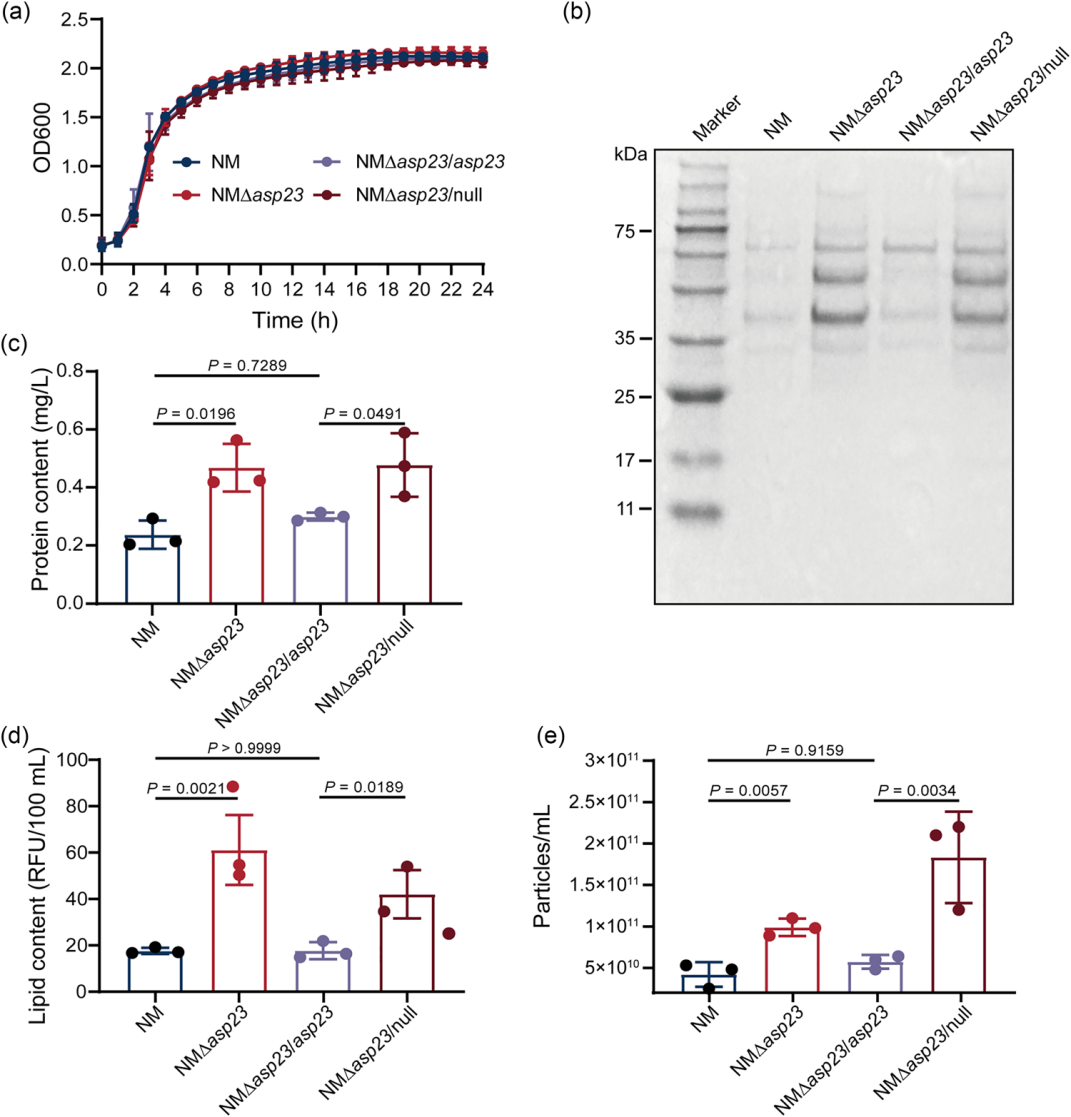
Increased MV production upon isogenic *asp23* deletion. (a) Bacterial growth profiling. *S. aureus* strain NM and derivatives (NMΔ*asp23*, NMΔ*asp23*/*asp23* NMΔ*asp23*/null) were cultivated in BHI broth in triplicate, and optical density at 600 nm for each culture was monitored hourly for 24 h. (b–e) MVs derived from stationary‐phase (16 h) cultures of *S. aureus* strain NM and derivatives (NMΔ*asp23*, NMΔ*asp23*/*asp23* NMΔ*asp23*/null) were compared by (b) SDS‐PAGE analysis, and quantified for (c) protein content, for (d) lipid content, and for (e) MV particle numbers. Representative image for SDS‐PAGE is shown from three independent experiments. Data are expressed as mean ± SD of three biological replicates and analyzed using one‐way ANOVA with Tukey's multiple comparisons test.

Since the Q225P mutation represses *asp23* expression and increases MV production, we hypothesized that an isogenic mutation in *asp23* would directly trigger MV biogenesis. To test this, *asp23* was seamlessly deleted from WT strain NM via homologous recombination to generate NMΔ*asp23*. The *asp23* was also provided *in trans* to obtain the complementary strain NMΔ*asp23*/*asp23*, with NMΔ*asp23*/null serving as a control. The absence of *asp23* did not affect bacterial growth kinetics (Figure 3a). SDS‐PAGE analysis for MVs showed that isogenic deletion of *asp23* greatly promotes MV formation as compared to WT NM, and the increased MVs can be reversed by complementing *asp23 in trans* (Figure 3b). The protein content of NMΔ*asp23*‐derived MVs was approximately 2‐fold greater than that of WT‐derived MVs (0.47 ± 0.03 vs. 0.24 ± 0.03 mg/L) (Figure 3c). Consistently, the lipid content of MVs derived from NMΔ*asp23* was 3.2‐fold higher than that from WT‐derived MVs (61.14 ± 12.31 RFU/100 vs. 17.63 ± 1.10 RFU/100 mL) (Figure 3d). Additionally, nanoparticle tracking analysis also revealed significantly increased particle numbers for MVs collected from NMΔ*asp23* in contrast to NM (Figure 3e). Furthermore, the release of MVs in the *asp23* deletion mutant was significantly diminished when *asp23* was complemented, as evidenced by quantitation of protein, lipid and MV particle numbers (Figure 3c–e). Taken together, we conclude that the isogenic mutation in *asp23* directly triggers MV biogenesis in *S. aureus* strain NM.

### 3.4 Absence of *asp23* results in weakened cell wall and enhanced autolysis in *S. aureus*

**FIGURE 4:**
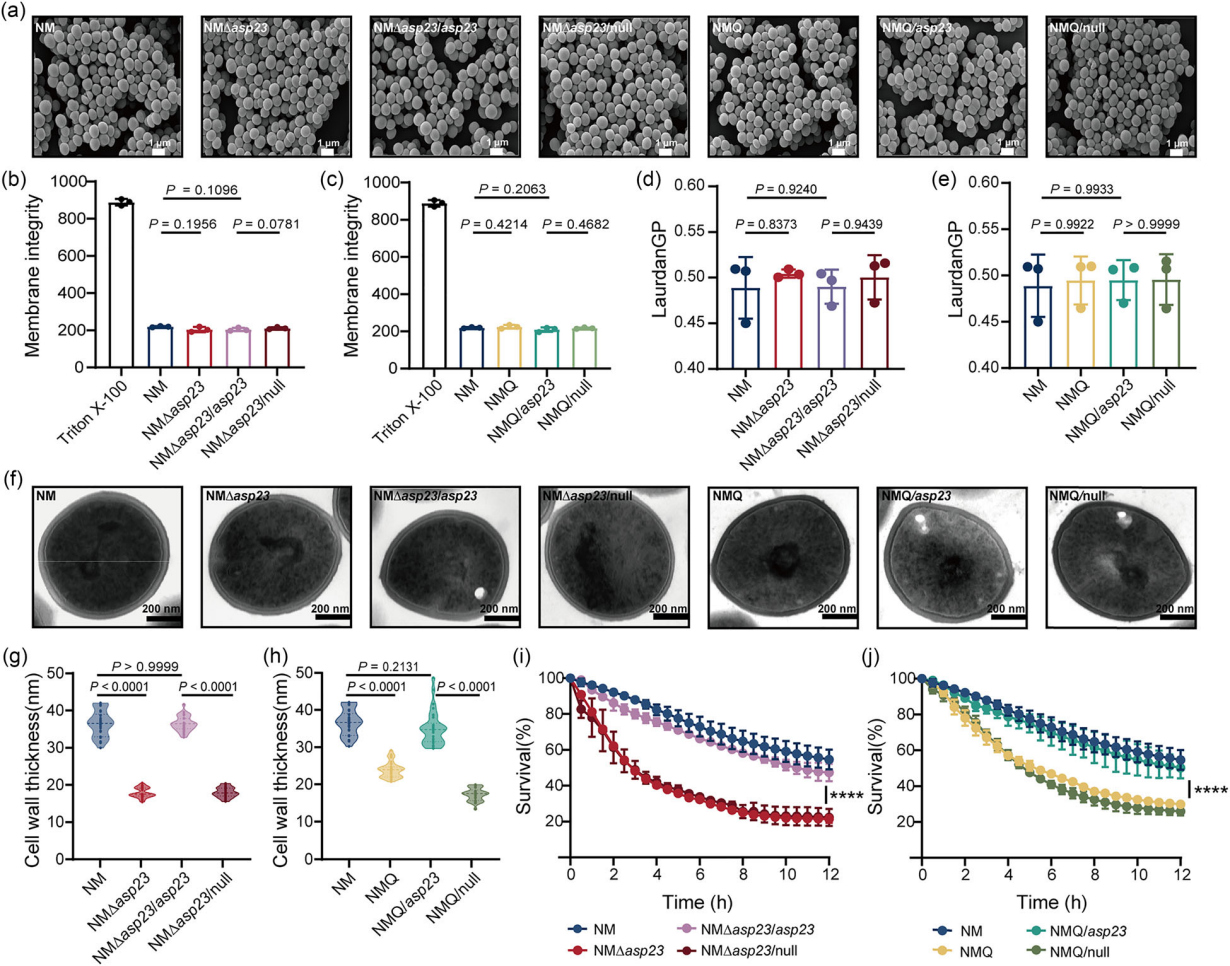
Deletion of *asp23* elicits weakened cell walls and enhanced autolysis. (a) Morphology of *S. aureus* cells collected from stationary‐phase (16 h) cultures was examined using SEM. Representative SEM images for strain NM and derivatives differing in *asp23* expression are displayed. Scale bar = 1 μm. (b, c) Assessment of cytoplasmic membrane integrity using PI staining for (b) NM, NMΔ*asp23* and derivatives differing in *asp23* expression, or for (c) NM, NMQ and derivatives differing in *asp23* expression. Bacterial suspension of NM that was pretreated with 1% Triton X‐100 for 1 h was used as a positive control. Data are shown as mean ± SD of three biological replicates. Statistical significance was analyzed by one‐way ANOVA with Tukey's multiple comparisons test. (d, e) Assessment of cytoplasmic membrane fluidity by Laurdan staining for *S. aureus* strains as the same in (b, c). Data are shown as mean ± SD of three biological replicates, and analyzed by one‐way ANOVA with Tukey's multiple comparisons test. (f–h) Cell wall thickness of *S. aureus* cells collected from stationary‐phase (16 h) cultures was determined by TEM. Representative TEM images for strain NM and derivatives differing in *asp23* expression are shown. Scale bar = 200 nm. Cell wall thickness was calculated from *n* = 30 individual cells from each strain, and each cell was randomly measured at 10 different points. Data are expressed as mean ± SD, and analyzed by one‐way ANOVA with Tukey's multiple comparisons test. (i, j) Triton X‐100‐induced autolysis of interested *S. aureus* strains was monitored in Tri‐HCl buffer, and the percentage of the initial OD600 was calculated as survival. Data are expressed as mean ± SD of three biological replicates, and analyzed by two‐way ANOVA, ^＊＊＊＊^ *P* < 0.0001.

Alkaline shock protein 23 (Asp23) is one of the most abundant proteins in the stationary‐phase of *S. aureus* growth, with approximately 25,000 molecules per cell (Maass et al., 2011). Asp23 is anchored in the cell membrane of *S. aureus* with the assistance of protein AmaP, and has been shown to regulate the *S. aureus* stress response to alkaline shock and to modulate resistance to the cell‐membrane‐targeting lipopeptide antibiotic daptomycin (Barros et al., 2019; Müller et al., 2014). To gain further insight into the Asp23‐mediated control of MV formation, we first determined whether cell envelope integrity is disturbed by *asp23* knockout. SEM analysis showed that both the WT NM and mutants (NMΔ*asp23*, NMQ and derivatives differing in *asp23* expression) exhibit identical and intact cellular morphologies (Figure 4a). Staining with the DNA‐binding dye PI, which can only penetrate damaged cell envelopes, demonstrated comparable membrane permeability between mutant NMΔ*asp23* and WT, as well as between NMQ and WT (Figure 4b,c). We also assessed membrane fluidity using the fluorescent probe Laurdan and found no significant difference in membrane fluidity in cells bearing the *asp23* deletion or the Q225P mutation, compared to that of WT (Figure 4d,e).

Interestingly, TEM showed that NMΔ*asp23* cell wall was significantly thinner, with an average thickness of 17.49 ± 1.30 nm, compared with that of WT (36.12 ± 3.44 nm) (Figure 4f,g). Moreover, the cell wall thickness was increased to the WT level for mutant NMΔ*asp23* when *asp23* was complemented *in trans*, which forms cells with an average cell wall thickness of 36.13 ± 1.99 nm (Figure 4g). Similar effects were also detected in mutant NMQ, in which cell walls have an average thickness of 23.17 ± 2.11 nm, and in NMQ bearing the *asp23 in trans*, where the cell walls are restored to 34.50 ± 4.58 nm (Figure 4f,h). As expected, the attenuated integrity of the cell wall affects autolysis, a major indicator of cell wall homeostasis. Autolysis increased significantly in both mutant NMΔ*asp23* and NMQ after treatment with Triton X‐100, and the effect was reversed by complementing *asp23 in trans* (Figure 4i,j). These results suggest that deletion or down‐regulation of *asp23* results in cell wall thinning and promotes autolysis, while having no effect on the integrity and fluidity of the cytoplasmic membrane.

### 3.5 Transcriptomic analysis reveals potential factors involved in *asp23*‐controlled MV biogenesis in *S. aureus*

**FIGURE 5:**
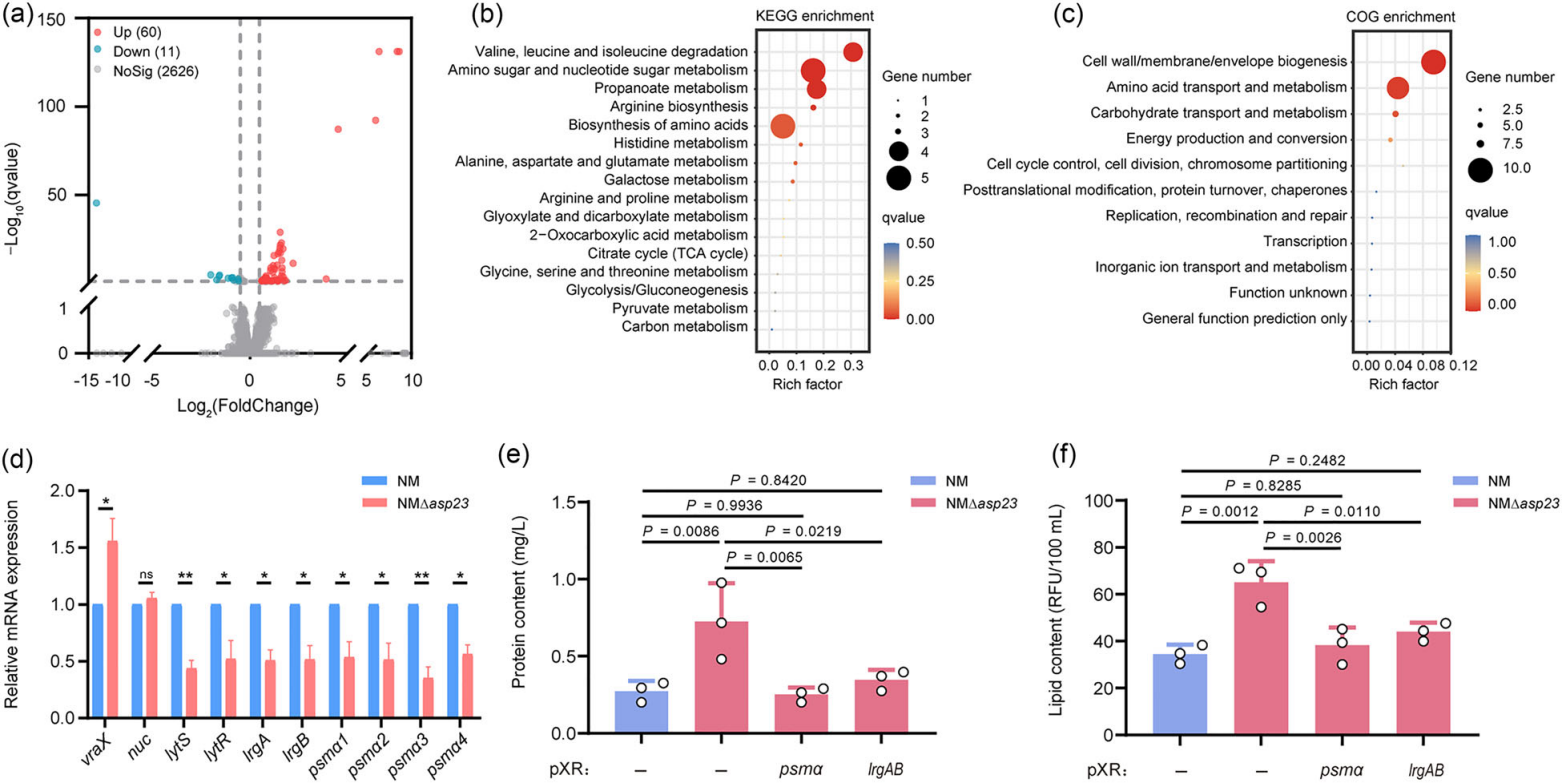
Deletion of *asp23* reduces expression of alpha‐type *psm* and *lrgAB* that contribute to Asp23‐controlled MV biogenesis. (a) RNA‐seq analysis showing DEGs in a comparison between *S. aureus* strain NM and NMΔ*asp23*. Down‐regulated DEGs in NMΔ*asp23* are represented by blue circles, and up‐regulated DEGs by red circles. Grey circles represent genes for which expression is not significantly changed. Dotted lines represent the DEG classification thresholds. (b, c) Up‐regulated DEGs in NMΔ*asp23* were subjected to KEGG enrichment analysis (b) or COG enrichment analysis (c). (d) Transcriptional levels of selected genes were measured in NM and NMΔ*asp23* using RT‐qPCR. *NWMN_0542* (*vraX*) that known to be positively regulated by *asp23* deletion and *nuc* that not regulated by *asp23* deletion were used as controls. Data are shown as mean ± SD for three biological replicates. Significance was determined using Student's *t*‐test. ^＊^ *P* < 0.05; ^＊＊^ *P* < 0.01; ns, not significant. (e, f) MVs were collected from stationary‐phase (16 h) cultures of *S. aureus* strain NM and NMΔ*asp23* that contains either the empty vector pXR or derivatives (pXR*psmα* and pXR*lrgAB*). Overexpression of *psmα* and *lrgAB* was induced by 0.5% (wt/vol) xylose during bacterial growth. MVs were quantified for (e) protein content and for (f) lipid content. Data are mean ± SD from three biological replicates and analyzed by one‐way ANOVA with Tukey's multiple comparisons test.

To investigate *asp23*‐mediated control of MV production, total cellular RNA was extracted from the WT and isogenic mutant NMΔ*asp23* and analyzed using high‐throughput sequencing to identify changes in the gene expression profiles. Genes were classified as differentially expressed if they exhibited a 1.5‐fold or greater change in expression, with a FDR *P*‐value < 0.05. A total of 71 DEGs were identified, including 60 up‐regulated and 11 down‐regulated genes in mutant NMΔ*asp23* relative to WT, respectively (Figure 5a, Table S3 in the Supplemental material). Functional enrichment analysis showed that the up‐regulated DEGs are significantly enriched in several metabolic‐related KEGG pathways, such as valine, leucine and isoleucine degradation, amino sugar and nucleotide sugar metabolism, propanoate metabolism, arginine biosynthesis, histidine metabolism, and alanine, aspartate, and glutamate metabolism (Figure 5b). COG enrichment analysis also showed two significantly enriched pathways for the up‐regulated DEGs, including cell wall/membrane/envelope biogenesis, and amino acid transport and metabolism (Figure 5c).

No significantly enriched pathways were identified by KEGG or COG analyses for the 11 down‐regulated DEGs. However, several down‐regulated DEGs have been implicated in cellular envelope maintenance that may affect MV biogenesis, including *lrgB* and four alpha‐type PSM‐coding genes (*psmα1*, *psmα2*, *psmα3* and *psmα4*) (Table S3). Previous studies demonstrated that *lrgB* and *lrgA* form a dicistronic operon, with *lrgB* encoding an antiholin‐like protein and *lrgA* encoding a murein hydrolase exporter, and elicit an inhibitory effect on the activity of murein hydrolases (Brunskill & Bayles, 1996; Groicher et al., 2000). Meanwhile, the two‐component system LytSR has been shown to positively regulate the expression of *lrgAB* and inhibits the peptidoglycan hydrolases, in which a *lytS* mutation promotes hydrolysis and autolysis of *S. aureus* (Brunskill & Bayles, 1996). In addition, PSMs, a group of small alpha helical, amphipathic peptides with surfactant‐like properties, have been confirmed to directly regulate MV biogenesis in *S. aureus*, wherein alpha‐type PSMs (particularly PSMα3) promote *S. aureus* MV release at low concentrations (less than 12.5 μg/mL), but destroy MVs at high concentrations (more than 12.5 μg/mL) as bacterial growth enters the stationary‐phase, indicating opposing effects on MV yield (Cheung et al., 2014; Schlatterer et al., 2018). Given that MVs were collected from stationary‐phase (16 h) cultures in our study, we therefore measured the expression levels for these eight genes (*lrgA*, *lrgB*, *lytS*, *lytR*, *psmα1*, *psmα2*, *psmα3* and *psmα4*) using RT‐qPCR. Simultaneously, *NWMN_0542* (*vraX*) (analogous to *SAOUHSC_00561* in *S. aureus* strain NCTC 8325) that known to be significantly up‐regulated in an *asp23* mutant (Müller et al., 2014), and *nuc* that identified with no significant change upon *asp23* deletion in our RNA‐seq analysis, were included serving as control. RT‐qPCR confirmed that all eight genes are significantly down‐regulated in mutant NMΔ*asp23* compared to WT (Figure 5d).

To further uncover the role of alpha‐type PSMs and LrgAB in Asp23‐controlled MV biogenesis, we constructed the transcriptional fusions in mutant NMΔ*asp23*, wherein the expression of *psmα* (comprising *psmα1* to *psmα4*) or *lrgAB* (including *lrgA* and *lrgB*) is controlled by a xylose‐inducible promoter. Overexpression of either *psmα* or *lrgAB* significantly reduced MV production in the *asp23* mutant, as demonstrated by quantitation of both the protein content and lipid content for MVs collected from stationary‐phase (16 h) cultures (Figure 5e,f). These results reveal that both alpha‐type PSMs and LrgAB contribute to hypervesiculation upon *asp23* deletion.

### 3.6 Properties and proteomic analysis of MVs generated by the *asp* 23 deletion mutant

**FIGURE 6:**
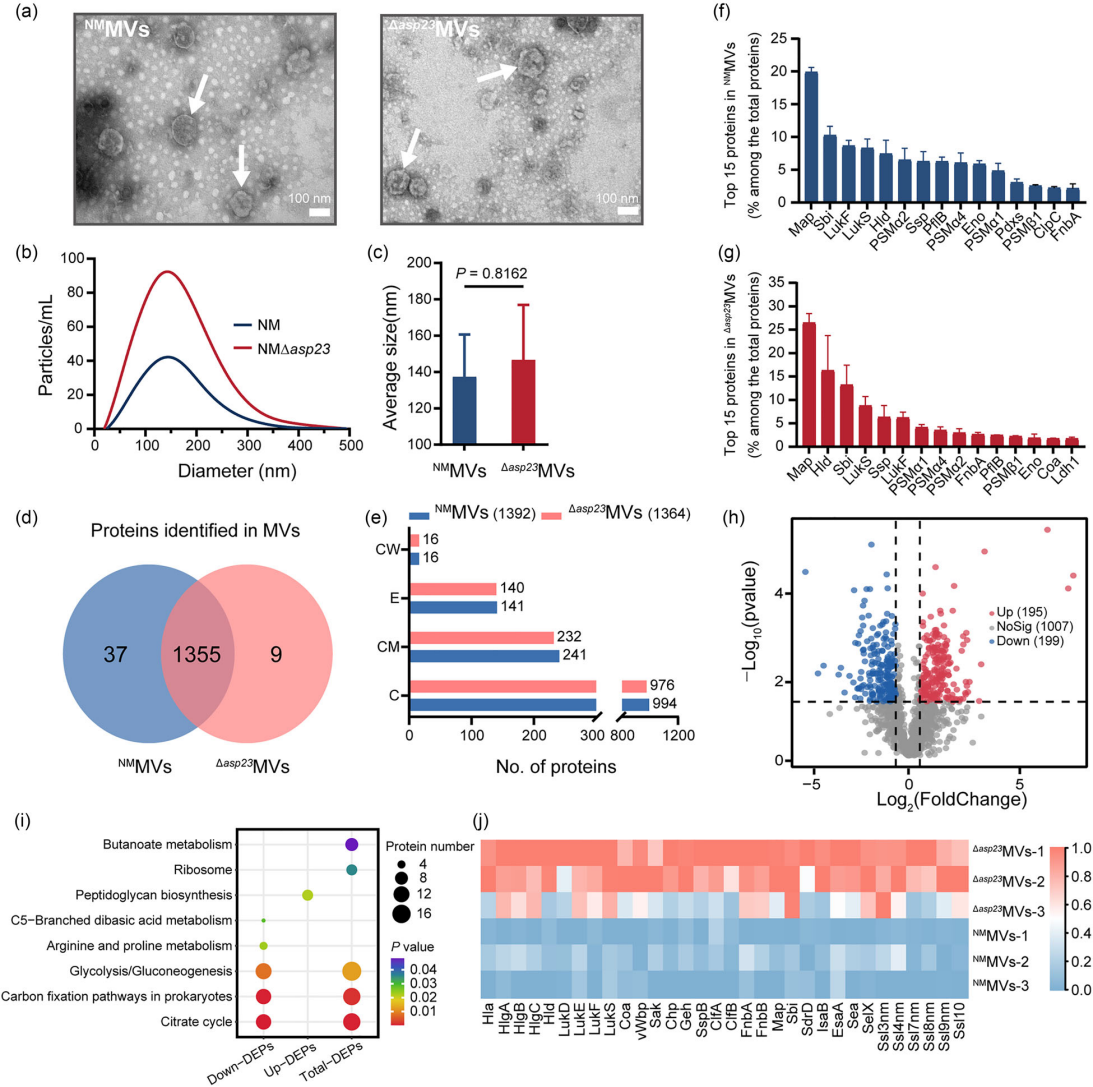
Characterization and proteomic analysis of MVs purified from NM and *asp23* deletion mutant. (a) MV morphology was observed by TEM. Representative TEM images of MVs derived from *S. aureus* strain NM and NMΔ*asp23* are shown. MVs are indicated with white arrows. Scale bar = 100 nm. (b, c) The size distribution (b) and the average size (c) of MVs generated by NM and NMΔ*asp23* were measured by nanoparticle tracking analysis. Representative size distribution of MVs is shown from three independent experiments. Data are expressed as mean ± SD of three biological replicates. Significance was determined using Student's *t*‐test. (d) Statistics of the total number of proteins identified in MVs derived from NM and NMΔ*asp23* using 4D‐label‐free LC‐MS/MS. (e) Statistics of the subcellular localization of proteins from purified MVs. C, cytoplasmic; CM, cell membrane‐associated; E, extracellular; CW, cell wall‐associated. (f, g) Relative abundance of the top 15 proteins identified in ^NM^MVs (f) and ^Δ^ *^asp23^* MVs (g), determined by label‐free quantity (LFQ) intensity. Data are shown as mean ± SD of three biological replicates. (h) DEPs detected by proteomic analysis in a comparison of ^NM^MVs and ^Δ^ *^asp23^* MVs. Down‐regulated DEPs in ^Δ^ *^asp23^* MVs are represented by blue circles, and up‐regulated DEPs by red circles. Grey circles represent genes for which protein levels are not significantly changed. (i) KEGG enrichment analysis for down‐regulated DEPs, up‐regulated DEPs, and total DEPs, respectively. Only significantly enriched pathways with a *P‐*value < 0.05 are shown. (j) Expression profiles for selected up‐regulated DEPs related to virulence in ^Δ^ *^asp23^* MVs compared to ^NM^MVs. Relative expression levels for each protein were normalized across samples based on LFQ intensity.

MV structure and composition in *S. aureus* can vary across lineages and even strains (Bitto et al., 2021; Wang et al., 2021). To determine the impact of *asp23* deletion on MV physiology, we purified MVs from *S. aureus* strain NM (designated ^NM^MVs) and NMΔ*asp23* (designated ^Δ^ *^asp23^* MVs) using OptiPrep density gradient ultracentrifugation. TEM revealed no noticeable morphological differences between the MVs, as both exhibited nano‐sized, spherical structures (Figure 6a). In addition, ^NM^MVs and ^Δ^ *^asp23^* MVs particles were similar in size distribution, with an average diameter of 137.48 ± 23.15 and 146.73 ± 30.24 nm, respectively (Figure 6b,c).

To examine MV composition and cargos in more detail, the purified MVs were analyzed by 4D‐label‐free liquid chromatography‐tandem mass spectrometry (LC‐MS/MS). A total of 1401 proteins were identified, of which 1355 proteins were found in both ^NM^MVs and ^Δ^ *^asp23^* MVs, 37 were detected only in ^NM^MVs, and 9 were specific for ^Δ^ *^asp23^* MVs (Figure 6d, Table S4 in the Supplemental material). Subcellular localization prediction of the proteins loaded in ^NM^MVs suggests that many are cytoplasmic (994, 71.4%), cell membrane‐associated (241, 17.3%), extracellular (141, 10.1%) and cell wall‐associated (16, 1.1%) (Figure 6e). The proteins associated with ^Δ^ *^asp23^* MVs exhibited an identical subcellular localization profile compared to ^NM^MVs (Figure 6e). The finding that cytoplasmic and membrane‐associated proteins were preferentially enriched, while cell wall‐associated proteins were much less abundant is consistent with previous studies (Chen et al., 2023; Uppu et al., 2023). Additionally, the intensity and relative proportion of the top 15 proteins identified in ^Δ^ *^asp23^* MVs and ^NM^MVs were quite similar, and proteins such as Map, Sbi, LukSF, Hld and PSMs were the most abundant molecules (Figure 6f,g).

By comparing protein levels in ^Δ^ *^asp23^* MVs and ^NM^MVs, 394 DEPs were identified, using fold change > 1.5 and *P* < 0.05 as classification thresholds. 195 DEPs were significantly up‐regulated, and 199 DEPs down‐regulated, in ^Δ^ *^asp23^* MVs (Figure 6h, Table S4). Functional enrichment analysis revealed that the citrate cycle was the most significant KEGG pathway involved in the down‐regulated DEPs, followed by carbon fixation, glycolysis/gluconeogenesis, arginine and proline metabolism, and C5‐branched dibasic acid metabolism (Figure 6i). In contrast, the up‐regulated DEPs were significantly enriched in the peptidoglycan biosynthesis pathway, in which several proteins were related to cell wall homeostasis (MurB, Pbp1, Pbp2, Pbp3 and Pbp4) (Figure 6i, Table S4). Nonetheless, functional enrichment analysis for the total 394 DEPs showed that the KEGG pathways of citrate cycle, carbon fixation, glycolysis/gluconeogenesis, ribosome and butanoate metabolism were significantly enriched, but not for peptidoglycan biosynthesis pathway (Figure 6i).

Previous studies have shown that deletion of *asp23* upregulates the cell wall stress response in *S. aureus* and promotes expression of the corresponding genes, such as the envelope stress marker *NWMN_0542* (analogous to *SAOUHSC_00561* in *S. aureus* strain NCTC 8325), *prsA* (encoding the peptidylprolyl isomerase) and *vraR* (encoding the response regulator) (Müller et al., 2014). These genes were also identified by RNA‐seq analysis and/or proteomic quantitation in our study (Tables S3 and S4). Interestingly, most of the up‐regulated DEPs in ^Δ^ *^asp23^* MVs are known virulence factors that are either secreted (such as hemolysin, enterotoxin, coagulase, leukocidin, staphylokinase and lipase) or cell wall‐associated (such as fibrinogen binding protein and IgG binding protein) (Figure 6j, Table S4). These factors may increase the toxicity of ^Δ^ *^asp23^* MVs and/or confer them with immunostimulatory effects, compared to ^NM^MVs.

## 4 DISCUSSION

Release of MVs is a common feature among eukaryotes, archaea and bacteria. In contrast to the well‐established mechanisms of vesiculation in Gram‐negative bacteria, the genetic determinants and regulation of MV biogenesis in Gram‐positive bacteria remain poorly understood. It has been increasingly recognized that global regulators play crucial roles in modulating bacterial MV generation by driving the expression of numerous genes. For example, deletion of *sigB*, a gene that encodes a transcriptional factor essential for the stress response, leads to a significant decrease in both the abundance and proteomic diversity of MVs in *L. monocytogenes* (Lee et al., 2013). The phosphorylation of Spo0A, a master regulator of sporulation, triggers MV formation in *Clostridium perfringens* (Obana et al., 2017). An inactivating mutation in the two‐component regulatory system CovRS increases MV production via a mechanism that is still unknown in *Streptococcus pyogenes* (Chiang‐Ni et al., 2023; Resch et al., 2016). Additionally, several species‐specific genes have been implicated in vesiculation, as in the case of *psmα* and *hld* in *S. aureus* (Wang et al., 2018; Wang et al., 2023) and *virR* in *M. tuberculosis* (Rath et al., 2013). These findings indicate that MV biogenesis in Gram‐positive bacteria is a complicated process, most likely regulated by a complex network of genetic determinants (Xu et al., 2023). Here, we demonstrate that a Q225P point mutation in SigB triggers MV production in *S. aureus* by repressing *asp23* expression. Consistently, the isogenic deletion of *asp23* also increases MV production, demonstrating the important roles played by *sigB* and *asp23* in modulating *S. aureus* MV biogenesis.

Generally, MV production begins with the budding of the cytoplasmic membrane and its subsequent transit through the cell wall, yielding MVs through the mechanisms of blebbing or bubbling cell death (Toyofuku et al., 2023). Factors that affect the integrity of the cytoplasmic membrane and/or the homeostasis of the cell wall can ultimately affect vesicle production. Differences in phospholipid content have been reported between cytoplasmic membranes and MVs in both *S. pyogenes* and *L. monocytogenes* (Coelho et al., 2019; Resch et al., 2016), and lipoproteins have been shown to influence the biogenesis and toxin content of *S. aureus* MVs by affecting membrane fluidity (Wang et al., 2020). Similarly, β‐lactam antibiotics such as flucloxacillin and ceftaroline increase *S. aureus* MV formation by compromising the cross‐linking of the peptidoglycan layer (Andreoni et al., 2019). In our study, we found that both deletion and down‐regulation of *asp23* resulted in a thinner cell wall. However, these modifications did not impair bacterial growth, or affect cytoplasmic membrane integrity and fluidity, as evidenced by direct SEM and TEM observation and fluorescent staining. These results suggest that Asp23‐controlled MV production in *S. aureus* may also occur via the blebbing mechanism.

In contrast, autolysis increased significantly in *S. aureus* cells with weakened cell walls. These effects were observed in both the mutant NMΔ*asp23* and NMQ strains, compared to WT. *S. aureus* encodes several peptidoglycan hydrolases including Atl and Sle1 (autolysin and N‐acetylmuramoyl‐L‐alanine amidase), which function in autolysis and cell wall renewal (Bose et al., 2012). Our RNA‐seq analysis revealed that *lrgB* was significantly repressed in mutant NMΔ*asp23*. Previous studies have shown that proteins LrgB and LrgA inhibit the activity of murein hydrolases, and that the two‐component system LytSR positively controls the expression of *lrgAB* and inhibits peptidoglycan hydrolase activity (Brunskill & Bayles, 1996; Groicher et al., 2000). Consistently, we found that *lytS*, *lytR*, *lrgA* and *lrgB* were all significantly repressed in the mutant NMΔ*asp23*, suggesting that the absence of *asp23* may trigger MV production through enhanced autolysis regulated by the LytSR‐LrgAB system. Additionally, we confirmed by both RNA‐seq and RT‐qPCR that *psmα1*, *psmα2*, *psmα3* and *psmα4*, which encode alpha‐type PSMs, were significantly down‐regulated when *asp23* was deleted. Interestingly, a previous study revealed that alpha‐type PSMs (particularly PSMα3) promote the release of *S. aureus* MVs at concentrations below 12.5 μg/mL, but destroy MVs at concentrations higher than 12.5 μg/mL^33^. The study also demonstrated that MVs are mostly isolated from bacterial cultures between 6 and 8 h of cultivation, but only small amounts of MVs are recovered in WT culture supernatants after 10 h of growth, likely due to increased concentrations of PSMα that secreted into the supernatants over time (Schlatterer et al., 2018). Since MVs were obtained from stationary‐phase (16 h) cultures in our study, the increased MV yield in NMΔ*asp23* may also is due to the decreased MV damage caused by lower concentrations of PSMα that secreted into stationary‐phase culture supernatants. Consistently, overexpression of either *psmα* or *lrgAB* significantly reduced MV production in *asp23* mutant, suggesting the important role of PSMα and LrgAB in Asp23‐controlled MV biogenesis.

Because *S. aureus* MVs carry specific biologically active cargos and play critical pathophysiological functions, they may have high value in a variety of biomedical and biotechnological applications (Gan et al., 2023). For example, vaccination of mice with *S. aureus* MVs containing immunostimulatory DNA, RNA and peptidoglycan elicits a strong humoral immune response and promotes the release of cytokines and chemokines by epithelial cells (Bitto et al., 2021). Our group previously developed a multivalent nanosized viral vaccine based on MVs derived from attenuated *S. aureus* strain RN4220 (^Δ^ *^agr^* MVs) that induces antibodies against all four serotypes of dengue virus (Yuan et al., 2018). We recently showed that ^Δ^ *^agr^* MVs loaded with hemolysin‐coregulated protein 1 (Hcp1) from *Burkholderia pseudomallei* can protect mice against acute melioidosis (Zhu et al., 2022). While these results are quite encouraging, MV yield is typically quite limited, which seriously constrains further development of the technology. In this study, we found that the Q225P mutation in SigB and deletion of *asp23* trigger *S. aureus* MV release. Both modifications appear to offer a route for engineering *S. aureus* strains with markedly enhanced MV yields. Nevertheless, proteomic analysis showed that MVs derived from mutant NMΔ*asp23* (^Δ^ *^asp23^* MVs) are particularly enriched in secreted and membrane‐associated virulence factors compared to ^NM^MVs, including hemolysin, enterotoxin and enterotoxin‐like toxin, coagulase, leukocidin, fibrinogen‐binding protein and IgG‐binding protein. Whether ^Δ^ *^asp23^* MVs exhibit enhanced toxicity and/or immunogenicity needs further exploration.

Taken together, our data demonstrate that a Q225P point mutation reduces the expression of *asp23* by impairing the binding of SigB to the *asp23* promoter. Deletion or repression of *asp23* triggers MV production via distinct pathways that are possibly involved in LrgAB‐controlled cell autolysis and PSMα‐mediated MV destruction. The results emphasize the significance of *sigB* and *asp23* in modulating *S. aureus* MV biogenesis and advance our understanding of vesiculation in *S. aureus*.

## AUTHOR CONTRIBUTIONS

Ming Li, Gang Li and Renjie Zhou designed the experiments. Jia Li and Keting Zhu conducted the experiments. Chao Li, Wei Huang, Xing Tian and He Yan participated in the mutant construction, MV characterization and parts of phenotypic assays. Yan Zhao, Jing Zhou and Xindi Gao provided reagents and analyzed the data. Keting Zhu and Gang Li wrote the manuscript. Ming Li and Xiancai Rao revised the manuscript.

## CONFLICT OF INTEREST STATEMENT

The authors declare no conflicts of interest.

## Supporting information

Supporting Information

Supporting Information

Supporting Information

Supporting Information
