# Supplementary material for: Alkaline shock protein 23 (Asp23)‐controlled cell wall imbalance promotes membrane vesicle biogenesis in Staphylococcus aureus
Source: J Extracell Vesicles. 2024 Aug 28;13(9):e12501. doi: 10.1002/jev2.12501 (PMC11350273; doi:10.1002/jev2.12501)
Supplement: Supplementary file 1 — Supporting Information [file JEV2-13-e12501-s004.docx]

**Table S1. Bacterial strains and plasmids used in this study**

| **Strains/plasmids** | **Description** | **Reference/source** |
| --- | --- | --- |
| **Strains**  *S. aureus* |  |  |
| RN4220 | NCTC 8325-4 derivative, an initial recipient for modification of plasmids prepared from *E. coli* | [1] |
| Newman | NCTC 8178, ST8/*agr*-I, a highly virulent strain that is extensively used in *S. aureus* animal models | [2] |
| NMQ  NMΔ*sigB* | Newman containing a Q225P mutation in *sigB*  Newman with *sigB* deletion | [3]  This work |
| NMQ/*asp23*  NMQ/null | NMQ complemented with *asp23*, Amp^r^, Cm^r^  NMQ complemented with plasmid pLI50, Amp^r^, Cm^r^ | This work  This work |
| NMΔ*asp23*  NMΔ*asp23*/*asp23*  NMΔ*asp23*/null  NM/pGFP*asp23*  NMQ/pGFP*asp23*  NM/pXR  NMΔ*asp23*/pXR  NMΔ*asp23*/pXR*psmα*  NMΔ*asp23*/pXR*lrgAB*  *E. coli*  Trans1-T1 | Newman with *asp23* deletion  NMΔ*asp23* complemented with *asp23*, Amp^r^, Cm^r^  NMΔ*asp23* complemented with plasmid pLI50, Amp^r^, Cm^r^  Newman with the GFP-based reporter, Amp^r^, Cm^r^  NMQ with the GFP-based reporter, Amp^r^, Cm^r^  Newman with plasmid pXR, Amp^r^, Cm^r^  NMΔ*asp23* with plasmid pXR, Amp^r^, Cm^r^  NMΔ*asp23* with operon *psmα* overexpressed, Amp^r^, Cm^r^  NMΔ*asp23* with operon *lrgAB* overexpressed, Amp^r^, Cm^r^  Cloning host for maintaining recombinant plasmids | This work  This work  This work  This work  This work  This work  This work  This work  This work  TransGen biotech |
| **Plasmids** |  |  |
| pBT2  pBT2∆*sigB*  pBT2∆*asp23* | Shuttle vector, temperature sensitive, Amp^r^, Cm^r^  pBT2-derived plasmid for *sigB* knockout, Amp^r^, Cm^r^  pBT2-derived plasmid for *asp23* knockout, Amp^r^, Cm^r^ | [4]  This work  This work |
| pLI50 | *E. coli*-*S. aureus* shuttle cloning vector, Amp^r^, Cm^r^ | [5] |
| pLI*asp23*  pGFP  pGFP*asp23*  pXR  pXR*psmα*  pXR*lrgAB* | pLI50-derived plasmid carrying *asp23* and its native promoter, Amp^r^, Cm^r^  Reporter plasmid with *gfp* expression controlled by S10 ribosomal gene promoter, Amp^r^, Cm^r^  pGFP-derived plasmid carrying the *asp23* promoter, Amp^r^, Cm^r^  Expression vector with a xylose-inducible promoter, Amp^r^, Cm^r^  pXR-derived plasmid for *psmα* overexpression, Amp^r^, Cm^r^  pXR-derived plasmid for *lrgAB* overexpression, Amp^r^, Cm^r^ | This work  [6]  This work  [6]  This work  This work |

Amp^r^, ampicillin resistant; Cm^r^, chloramphenicol resistant

**References**

1. Berscheid A, Sass P, Weber-Lassalle K, Cheung AL, Bierbaum G. Revisiting the genomes of the *Staphylococcus aureus* strains NCTC 8325 and RN4220. *Int J Med Microbiol*. 2012; 302(2): 84-87.

2. Duthie ES, Lorenz LL. Staphylococcal coagulase; mode of action and antigenicity. *J Gen Microbiol*. 1952; 6(1-2): 95-107.

3. Qiao L, Yang Y, Zhu K, *et al*. The Q225P Mutation in SigB Promotes Membrane Vesicle Formation in *Staphylococcus aureus*. *Curr Microbiol*. 2022; 79(3): 81.

4. Brückner R. Gene replacement in *Staphylococcus carnosus* and *Staphylococcus xylosus*. *FEMS Microbiol Lett*. 1997; 151(1): 1-8.

5. You Y, Xue T, Cao L, Zhao L, Sun H, Sun B. *Staphylococcus aureus* glucose-induced biofilm accessory proteins, GbaAB, influence biofilm formation in a PIA-dependent manner. *Int J Med Microbiol*. 2014; 304(5-6): 603-612.

6. Shang W, Rao Y, Zheng Y, *et al*. β-Lactam Antibiotics Enhance the Pathogenicity of Methicillin-Resistant *Staphylococcus aureus* via SarA-Controlled Lipoprotein-Like Cluster Expression. *mBio*. 2019; 10(3): e00880-19.
