# Supplementary material for: Alkaline shock protein 23 (Asp23)‐controlled cell wall imbalance promotes membrane vesicle biogenesis in Staphylococcus aureus
Source: J Extracell Vesicles. 2024 Aug 28;13(9):e12501. doi: 10.1002/jev2.12501 (PMC11350273; doi:10.1002/jev2.12501)
Supplement: Supplementary file 2 — Supporting Information [file JEV2-13-e12501-s002.docx]

**Table S2. Primers used in this study**

| **Primers** | **Sequences (5’–3’)** | **Description** |
| --- | --- | --- |
| upasp23-F | AGTGCAGCGGAATTCGAGCTCCGTAAAAAAGAACTAAGATTAAGAAGTTCAAA | For amplification of the left region of *asp23*  For amplification of the left region of *asp23* |
| upasp23-R | AGGAGAATCTATTGTTATTCGATAAAAAGGGCTTGG |  |
| downasp23-F | TTATCGAATAACAATAGATTCTCCTTTTACTTGTTAATTTTTATATTTTTT | For amplification of the right region of *asp23*  For amplification of the right region of *asp23* |
| downasp23-R | CCTGCAGGTCGACTCTAGAGGATCCAACTAGGGAATATATTACTAAATGGTACTTCA |  |
| asp23-F | CAAGCATACGACAATCAAACTG | For identification of *asp23* deletion  For identification of *asp23* deletion |
| asp23-R | TCTTTCTTGGTTATTGTTTTCGT |  |
| check-F | ATAAAAGAAGATGGAGATAGCGA | For verification of *asp23* deletion  For verification of *asp23* deletion |
| check-R | TCGCTTATAAAATCTTTGAGTGA |  |
| pLIasp23-F | GAGGCCCTTTCGTCTTCAAGGGCTAACAATCATAACCAAAAC | For complementation of *asp23*  For complementation of *asp23*  For amplification of *asp23* promoter  For amplification of *asp23* promoter  For overexpression of *psmα*  For overexpression of *psmα*  For overexpression of *lrgAB*  For overexpression of *lrgAB*  EMSA  EMSA  EMSA  EMSA  EMSA  EMSA  EMSA  EMSA  RT-qPCR  RT-qPCR  RT-qPCR  RT-qPCR  RT-qPCR  RT-qPCR  RT-qPCR  RT-qPCR  RT-qPCR  RT-qPCR  RT-qPCR  RT-qPCR  RT-qPCR  RT-qPCR  RT-qPCR  RT-qPCR  RT-qPCR  RT-qPCR  RT-qPCR  RT-qPCR  RT-qPCR  RT-qPCR |
| pLIasp23-R  pGFPasp23-F  pGFPasp23-R  pXRpsmα-F  pXRpsmα-R  pXRlrgAB-F  pXRlrgAB-R  Pasp23-F  Pasp23-R  Biotin-Pasp23-F  Biotin-Pasp23-R  Biotin-mutant-F  Biotin-mutant-R  Biotin-PsaeR-F  Biotin-PsaeR-R  gyrA-F  gyrA-R  vraX-F  vraX-R  nuc-F  nuc-R  lytS-F  lytS-R  lytR-F  lytR-R  lrgA-F  lrgA-R  lrgB-F  lrgB-R  psmα1-F  psmα1-R  psmα2-F  psmα2-R  psmα3-F  psmα3-R  psmα4-F  psmα4-R | TGCATGCCTGCAGGTCGACTTTATTGTAAACCTTGTCTTTCTTG  CAAGAATTCGAGCTCGGTACCGGCTAACAATCATAACCAAAACG  GAAAAGTTCTTCTCCTTTAATCATTCTAGACATAATAGATTCTCCTTTTACTTGTTAATTTTTATATT  AGGAGGAATATTATGGGTACCATGGGTATCATCGCTGG  GCAGGTCGACTCTAGAGGATCCTTATTTTGCGAAAATGTCGATAATTGC  AGGAGGAATATTATGGGTACCATGGTCGTGAAACAACAAAAAG  GCAGGTCGACTCTAGAGGATCCTTAGAAGAATATTGCTACAAAGACAGG  TGGTCCACGAGTGTTGTAAG  TCGTATGCTTGTTTTGCTTT  TGGTCCACGAGTGTTGTAAG  TCGTATGCTTGTTTTGCTTT  GCCAGTACGTAAGTTAGAAG  GCTTACGGTCTTTCATTATG  TCACCTCTGTTCTTACGACCTC  CCAGTCATCGCTAACAATAC  TGGTTCAATGGATGGAGATGGC  AGACTGACGGCTCTCTTTCA  ATATGATCTATATCGTCTTGTAAT  TTATCGACAGTATCACCAT  GTAGCCATCATTATTGTAGGT  GTATCACCATCCACAACTC  AGAAGGATTGAATAGAGAAT  TTGTTGGTATGTGATGAT  AAGATGATAATAACGCAAGTG  TAACGATTCAATGGCTCTG  GTTAAGTTAGGCGAAGTC  ACGATTATTAGTCCAATGATT  CATCGGAGGTATTGGTATCG  GTAGTTGCTGCTTGAGGTAA  TTATTTACCAGTGAATTGTTCGT  ATGGGTATCATCGCTGGCATC  TTACTTACCAGTGAATTTCTCAT  ATGGGTATCATTGCAGGAATCT  TTAGTTGTTACCTAAAAATTTAC  ATGGAATTCGTAGCAAAATTAT  TTATTTTGCGAAAATGTCGATAT  ATGGCTATTGTAGGTACTATCAT |  |
